# Therapeutic effect of dental pulp stem cell transplantation on a rat model of radioactivity-induced esophageal injury

**DOI:** 10.1038/s41419-018-0753-0

**Published:** 2018-07-03

**Authors:** Chunwei Zhang, Yichi Zhang, Zhenning Feng, Feifei Zhang, Zishuai Liu, Xiaoli Sun, Mengting Ruan, Mingna Liu, Shizhu Jin

**Affiliations:** Department of Gastrointestinal and Hepatology, The Second Affiliated Hospital, Harbin Medical University, Heilongjiang Harbin, China

## Abstract

Dental pulp stem cell (DPSC) transplantation has been demonstrated to promote the regeneration and repair of tissues and organs and is a potentially effective treatment for radioactive esophageal injury. In this study, to explore the therapeutic effects of DPSCs on acute radiation-induced esophageal injury, DPSCs were cultured and transplanted into rats with acute radioactive esophageal injuries induced by radioactive ^125^I seeds in vivo. In the injured esophagus, PKH26-labeled DPSCs co-localized with PCNA, CK14, CD71, and integrin α6, and the expression levels of these four makers of esophageal stem cells were significantly increased. After DPSC transplantation, the injured esophagus exhibited a greater thickness. In addition, the esophageal function and inflammation recovered faster. The results demonstrated that transplanted DPSCs, which trans-differentiated into esophageal stem cells in vivo, could repair the damaged esophageal tissue.

## Introduction

Currently, chemoradiotherapy is the established standard treatment for locally advanced tumors of the head, neck, and lung. However, severe toxicities, such as acute radioactive esophageal injury, can develop within 3 weeks of radiation therapy and often cause unexpected complications^1,2^. Although natural radioprotectors are clinically used to prevent radiation injury, ionizing radiation injuries are not completely avoided^3^. Repeated ionizing radiation causes dysphagia and odynophagia, which may lead to weight loss, when radiation dosages exceed 30 Gy^1–4^. Although acute radioactive esophageal injury is usually self-limited, severe esophageal injury can greatly lengthen the treatment period through additional hospitalization, esophageal ulceration, and clinical symptoms that include difficulty in swallowing, odynophagia, and substernal pain. Late-onset damage includes esophageal stricture, sclerosis, and tracheoesophageal fistula, which seriously impact a patient’s quality of life and long-term survival^5^. Acute radioactive esophageal injury is generally treated symptomatically with chemical agents^6,7^. Some of these agents, including amifostine, manganese superoxide dismutase-plasmid liposome, glutamine, recombinant human granulocyte-macrophage colony-stimulating factor and epidermal growth factor, have been reported to relieve radiation injuries in clinical and preclinical settings^8,9^. Recently, research interest in stem cell (SC) transplantation to treat tissues and organs damage has greatly increased. Several studies on the use of mesenchymal cells derived from tissues and organs have been published^10–12^. Furthermore, the use of isolated progenitor cells or SCs for regenerating irradiation-damaged tissues has achieved great progress in recent years. As shown in the study by Epperly et al.^13^, the injection of bone marrow SCs into the mouse esophagus promoted the healing of the injured esophageal tissue, and localized cells with homing capacities could undergo unlimited proliferation in the irradiation-injured recipient esophagus. Compared to bone marrow SCs and other SCs, dental pulp SCs (DPSCs) have additional advantages. DPSCs, a type of mesenchymal cell, have high proliferative capacity and can differentiate into osteoblasts, odontoblasts, adipocytes, neuronal cells, vascular cells, muscular cells, and epithelial cells^14–17^. The harvesting of dental SCs from extracted teeth has significant benefits compared with the harvesting of other SCs and other adult SCs, which require more invasive procedures that usually involve pain and the risk of adverse events. DPSCs are easily harvested from wisdom teeth, which are extracted worldwide and disposed of as medical waste; consequently, the study and application of DPSCs involve minimal ethical issues^18–20^. Moreover, DPSCs have been shown to be an efficient cell source for the treatment of many diseases^21,22^. Therefore, DPSCs provide an alternative ancestral cell source for regenerating the esophageal tissue via cell banking and could become a potential therapy for the treatment of radioactive esophageal injury.

The objective of this study was to evaluate the effects of DPSC implantation on the treatment of acute radioactive esophageal injury. We established an acute radioactive esophageal injury model to determine the effects of DPSC transplantation on esophageal tissue regeneration. ^125^I seeds were used to irradiate the esophageal tissue in this model; the ^125^I seeds were placed in a disposable ureteral catheter and inserted into the esophageal lumen. In the present study, the model was induced by ^125^I seeds in vivo, differing from the previous in vitro methods. To the best of our knowledge, the method used in the present study is the first of its kind to be described.

## Results

### Verification of the ^125^I seed-induced radiation injury in the esophagus

**Fig. 1:**
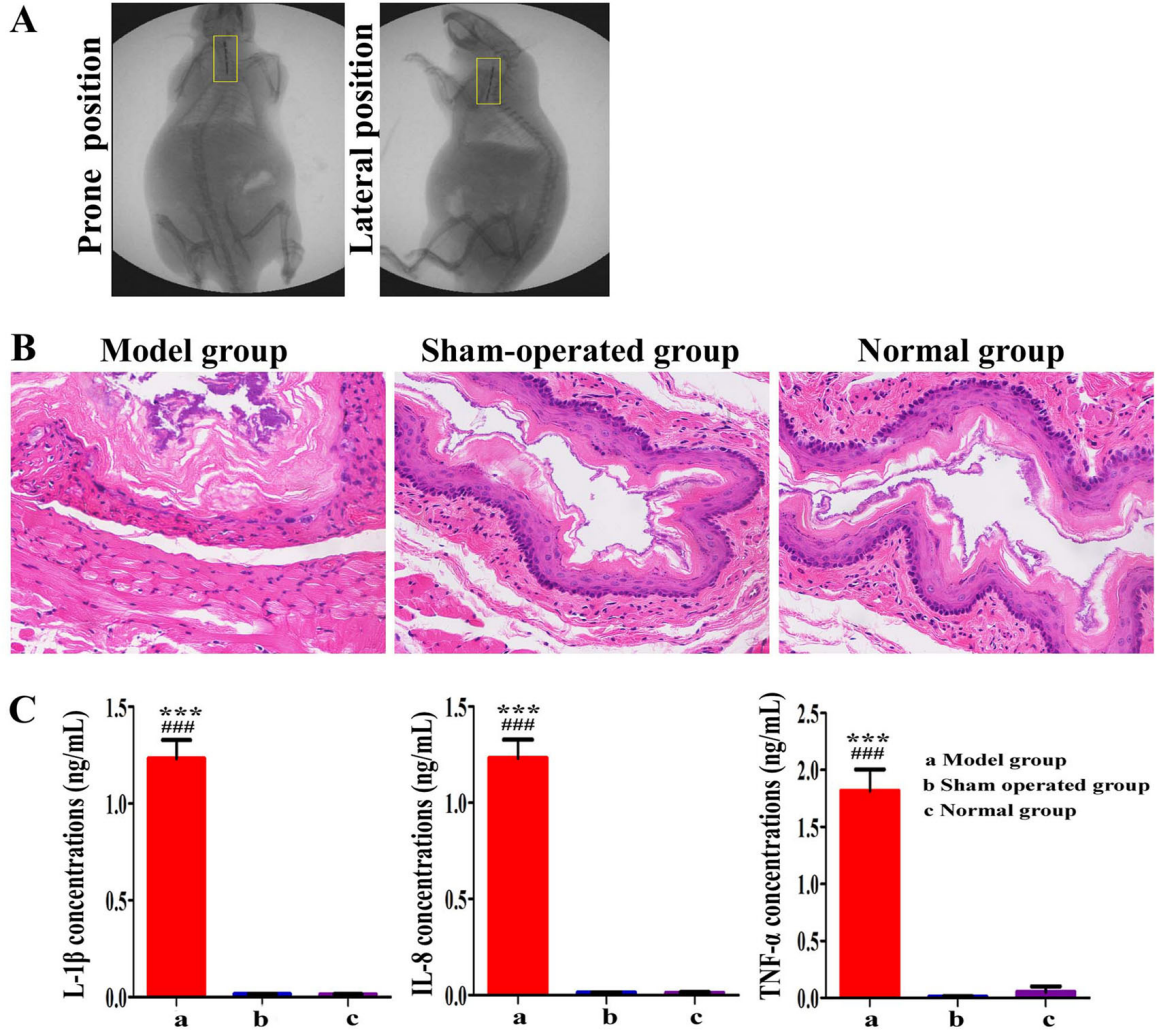
Evaluation of the model. **a** X-ray revealed that the ^125^I seed strain lined up in the esophageal lumen from the prone and lateral position. **b** Histological images showing esophageal tissue from the model, sham-operated and normal groups. The polygon cells and basal cells of the mucosa were arranged in a disorderly manner. Moreover, fractures, defects. and blood vessel congestion were observed. The lamina propria and submucosa were infiltrated by neutrophils and lymphocytes in the model group. However, the esophageal epithelium was well organized in the sham-operated and control groups (×100). **c** TNF-α, IL-1β, and IL-8 concentrations were significantly increased in the model group compared with those in the control and sham-operated groups on the fourth day after radiation (*P* < 0.0001). Moreover, there were no significant differences between the control and sham-operated groups (*P* > 0.05). *P < 0.05, **P < 0.001, ***P < 0.0001, the model group compared with the sham-operated group. ^#^*P* < 0.05, ^##^*P* < 0.001, ^###^*P* < 0.0001, the model group compared with the normal group

^125^I seeds were successfully placed in a predetermined location in the esophageal lumen. Using the X-ray positioner, we observed ^125^I seeds lined up in the esophageal lumen along the long axis (Fig. 1a). The vertical distance between the esophageal lumen and each ^125^I seed was 0.05 mm in this experimental model, and the center point of the seed dose rate was 43 cGy/h, according to the formula ${\dot{D}}$(*r*, *θ*) = *N*Σ${\dot{D}}$(*r*_i_, *θ*_i_)^23,24^. Using the naked eye, we also observed that the esophageal mucosa became swollen after 5 days of exposure to radiation in the model group. The polygon cells and basal cells of the mucosa showed a disordered arrangement. Moreover, the fractures, defects, and blood vessel congestion had noticeably increased. The lamina propria and submucosa were infiltrated by inflammatory cells containing neutrophils and lymphocytes, and the thickness of the esophageal epithelium was obviously reduced. In the normal and sham-operated groups, the basal compartment of the rat esophageal epithelium consisted of a single row of densely packed cuboidal cells whose oval nuclei were oriented toward the lumen and were deeply basophilic. The suprabasal compartment included multiple layers of polyhedral cells with spherical nuclei, above which existed a granular layer that was equal to the esophageal epithelial surface of the degenerating nuclei. The lamina propria contained collagen fibers composed of fibroblasts, and the muscular layer contained internal circular muscles and external longitudinal muscles (Fig. 1b). The inflammatory cytokine levels of the rats in the model, sham-operated and control groups were compared. Compared with the normal and sham-operated groups on the fourth day after radiation, the model group exhibited significantly higher interleukin-1β (IL-1β), IL-8, and tumor necrosis factor-α (TNF-α) concentrations (*P* < 0.0001), and significant differences were not observed between the normal and sham-operated groups (*P* > 0.05) (Fig. 1c).

### Characterization of the isolated DPSCs

**Fig. 2:**
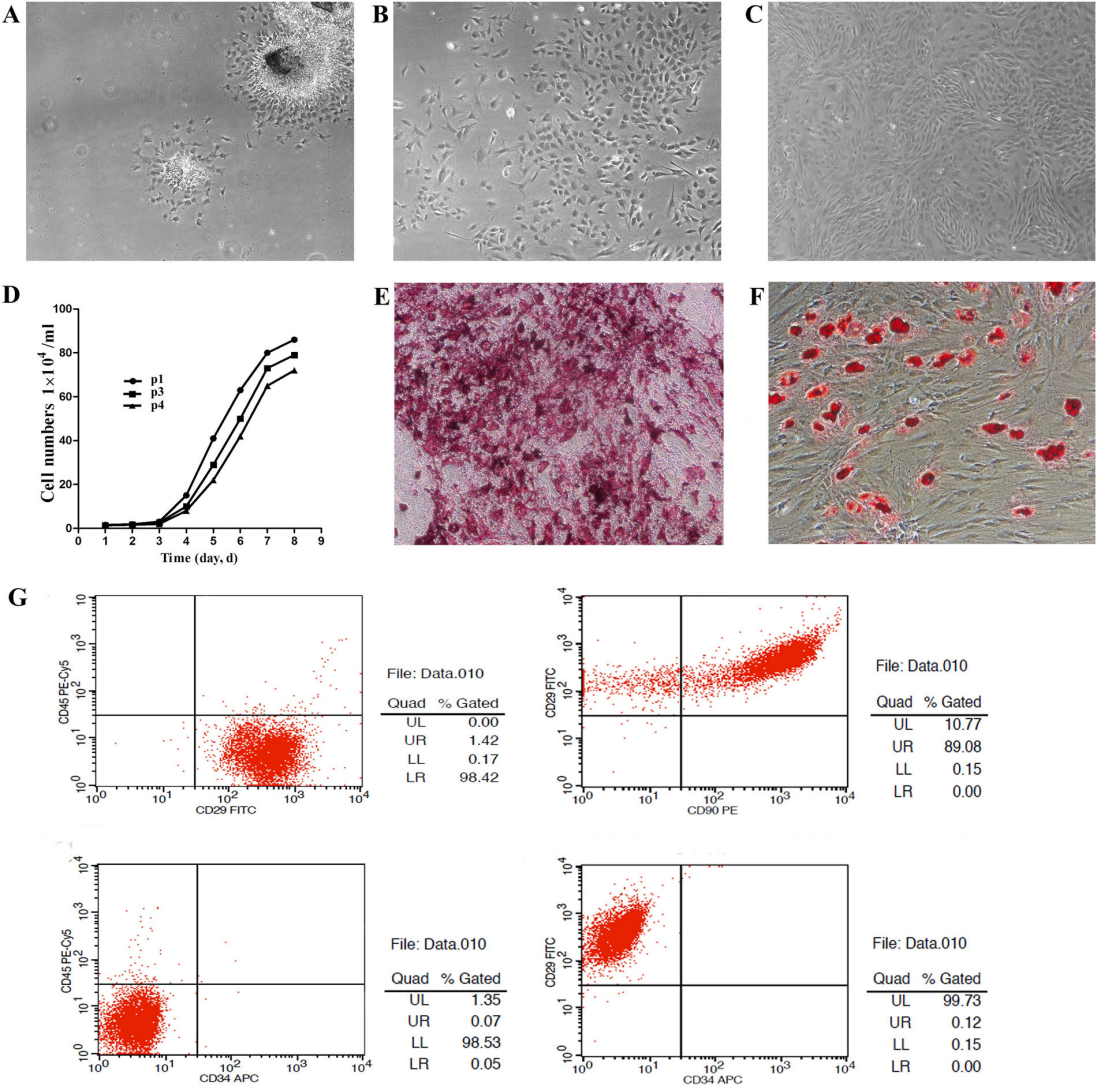
Morphological features of DPSC growth and flow cytometric analyses with DPSC markers. **a** Primary DPSCs assumed a fibroblast-like appearance on the first day (×40). **b** A large number of spindle cells attached to the culture dish on the fourth day (×40). **c** The majority (80–90%) of the DPSCs were confluent after 2 weeks (×40). **d** On days 1–3, the P1, P3, and P4 DPSCs proliferated slowly, and the cells appeared to proliferate rapidly from day 4, entering the logarithmic phase. The increase in the number of cells then slowed gradually, reaching the plateau phase on day 8. **e**, **f** The DPSCs differentiated into the osteogenic and adipogenic lineages (×100). **g** A total of 99.85% of the cells reacted with anti-rat CD29; 89.08% of the cells reacted with anti-rat CD90; 1.42% of the cells reacted with anti-rat CD45; and 0.12% of the cells reacted with anti-rat CD34

The DPSCs isolated from the incisors were cultured in dishes, early passage (P0) cells appeared triangular, polygonal, or spindle shaped when they began to stick to the bottom of the dish and form spindles (Fig. 2a), most of which were present after 4 days, exhibiting radial or spiral growth (Fig. 2b). Colony formation was observed between 5 and 6 days, and the cells appeared to confluence at 7–8 days. The DPSCs were removed from the petri dishes using trypsin, transferred into flasks, and amplified for the following 2 weeks (Fig. 2c).

The P1, P3, and P4 DPSCs in the latent phase proliferated slowly on days 1–3. SC colonies were observed on day 3, and the cells appeared to proliferate rapidly from day 4 onward and entered the logarithmic phase. The cell number then slowed down gradually, reaching the plateau phase on day 8 (Fig. 2d). According to the growth curve, P3 cells were selected.

Osteogenic differentiating cultures were determined based on a positive red area of mineralized matrix stained with alizarin red (Fig. 2e). Adipogenic differentiation was determined by the presence of lipid droplets stained red with Oil Red O (Fig. 2f).

Fluorescence-activated cell sorting (FACS) analysis was used to determine the expression levels of the specific DPSC antigen markers. In total, 99.85% of the expanded cells expressed CD29, and 89.08% of the cells expressed CD90. In contrast, 1.42% expressed CD45, a hematopoietic cell and leukocyte marker^25^, and only 0.12% expressed CD34, a dental pulp progenitor cell antigen that also appears on some epithelial cells and fibroblasts (Fig. 2g)^26,27^. Thus, the dental pulp cells extracted from the incisors of the Sprague–Dawley (S–D) rats were mostly DPSCs.

### Colocalization of the transplanted DPSCs in the esophagus

**Fig. 3:**
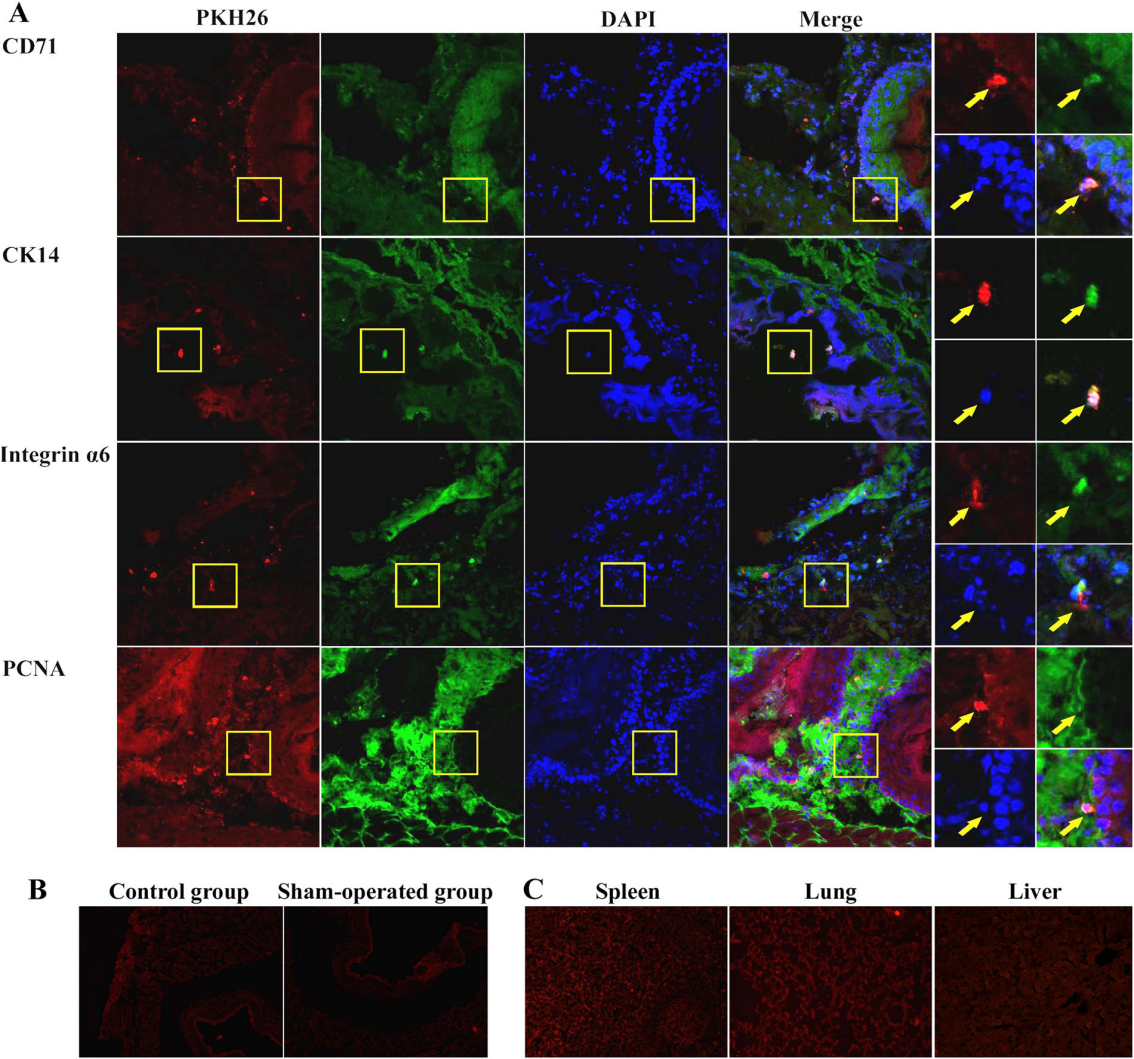
Colocalization of the transplanted DPSCs in the esophagus. **a** PKH26-labeled cells (red) co-localized with CD71, CK14, integrin α6, and PCNA (green) in the esophageal epithelium after transplantation. The cell nuclei were labeled with DAPI (blue) (×400 and ×800). **b**, **c** Few PKH26-positive cells were found in the control group, sham-operated group, and other organs, including spleen, lung, and liver, in the DPSC group (×100)

The transplanted DPSCs’ potential to proliferate and trans-differentiate into esophageal cells was evaluated for 4 weeks after cell transplantation. Some PKH26-positive cells appeared to colocalize with CD71 by 4 weeks post transplant. The PKH26-positive cells also co-expressed CK14, integrin α6, and PCNA (Fig. 3a). Many triple-labeled cells were located in pairs or small clusters of proliferating and differentiating cells. Furthermore, few PKH26-positive cells were found in the control group, the sham-operated group and other organs, including the spleen, lung, and liver, in the DPSC group (Fig. 3b).

### The characterization of DPSCs as proliferative and trans-differentiated cells

**Fig. 4:**
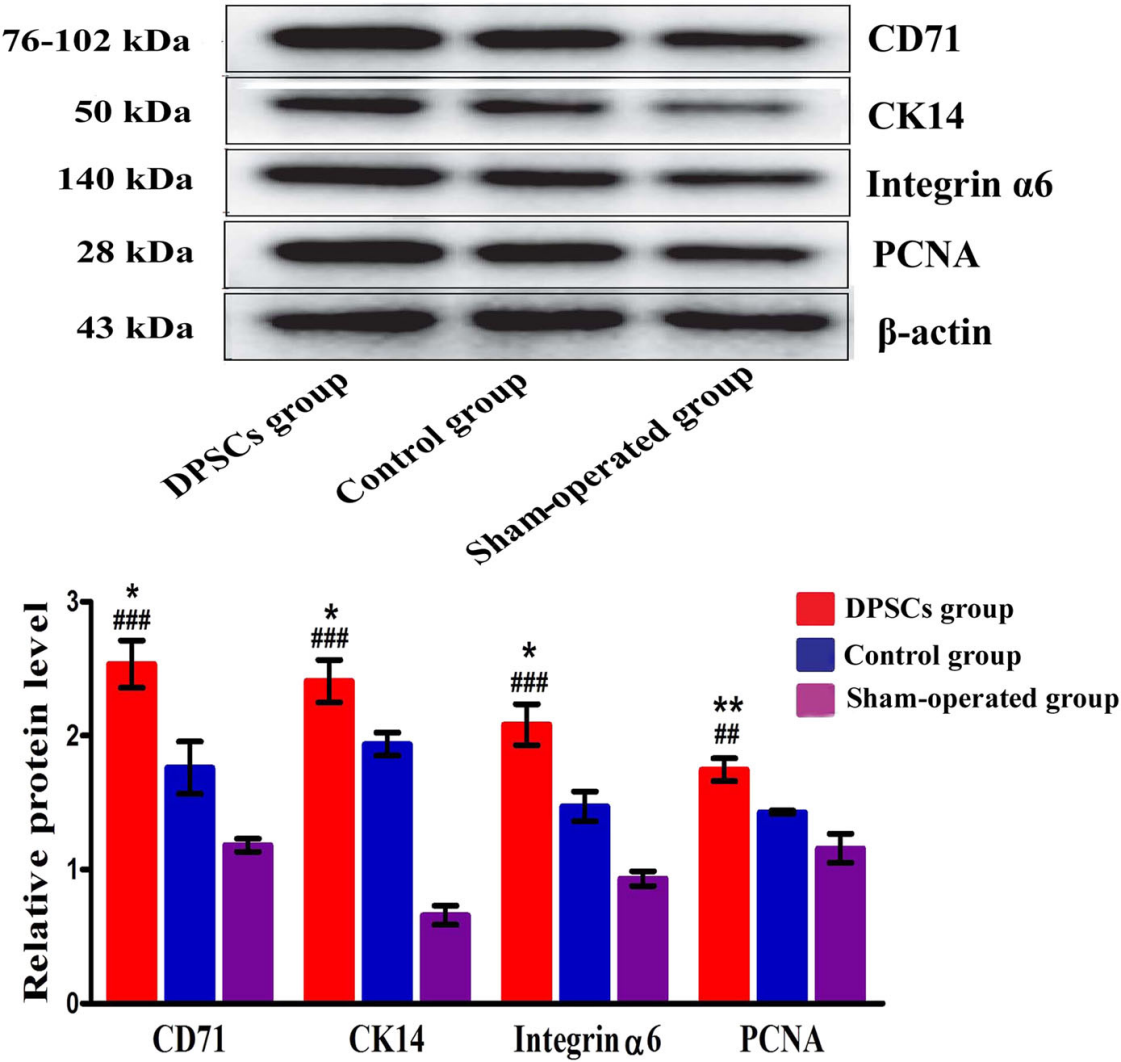
Protein expression levels of proliferative and trans-differentiated cell markers. The protein expression levels of CD71, CK14, integrin α6, and PCNA significantly increased in the DPSC group compared with those in the control and sham-operated groups. *P < 0.05, **P < 0.001, ***P < 0.0001, the DPSC group compared with the control group. ^#^*P* < 0.05, ^##^*P* < 0.001, ^###^*P* < 0.0001, the DPSC group compared with the sham-operated group

**Fig. 5:**
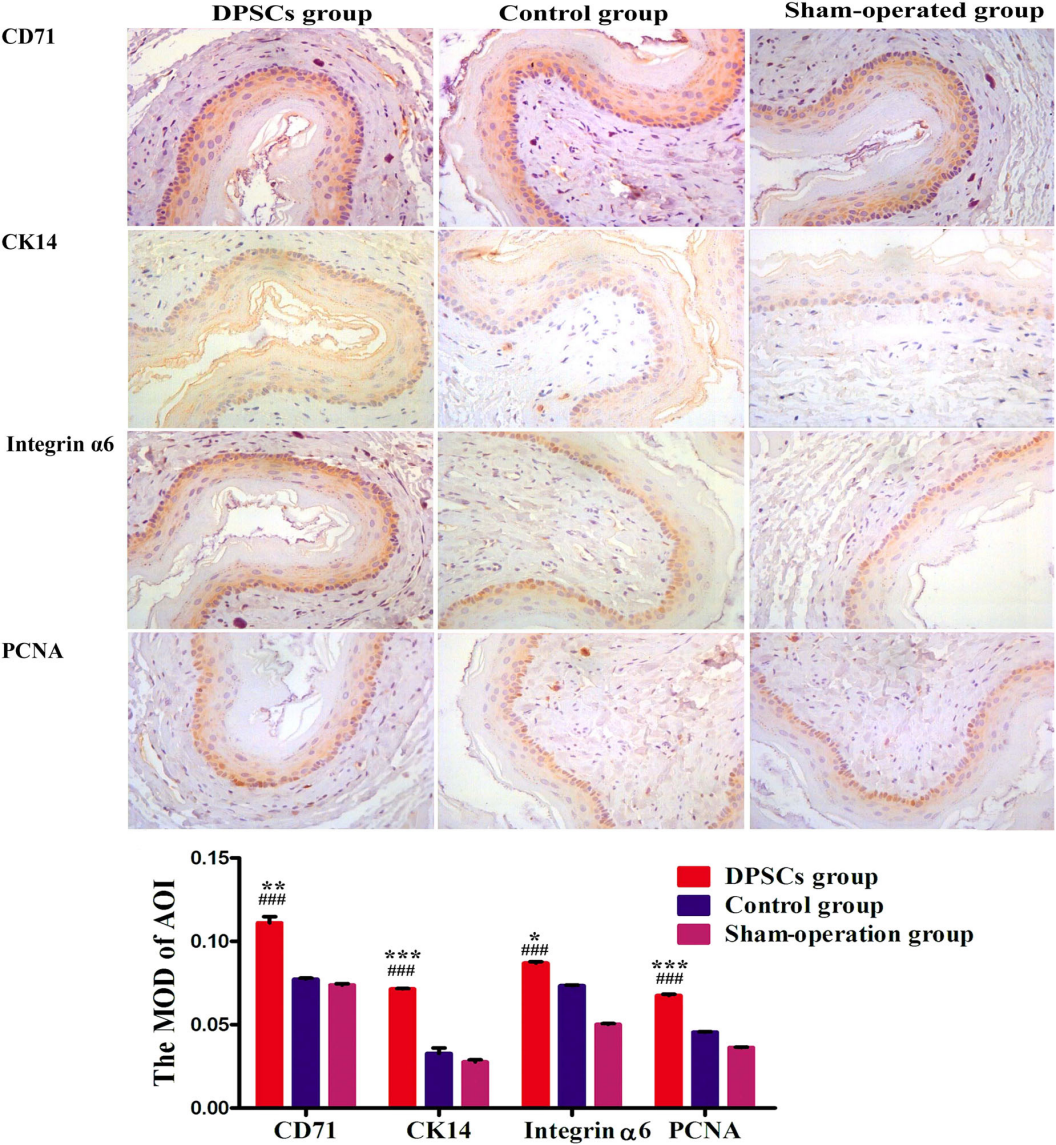
The MODs of the AOIs of proliferative and trans-differentiated cell markers. The MODs of the AOIs of CD71, CK14, integrin α6, and PCNA in the DPSC group were significantly higher than those in the control and sham-operated groups (×400)

Esophageal tissues from rats in all three groups were examined using Western blotting (WB) and immunohistochemistry to assess the proliferation and trans-differentiation of DPSCs into esophageal cell-like cells in the injured esophagus. We performed a quantitative analysis of the amounts of CD71, CK14, integrin α6, and PCNA in the DPSC, control, and sham-operated groups by measuring these protein levels and the mean optical density (MOD) of the area-of-interest (AOI). The levels of cell surface marker proteins in the DPSC group were significantly higher than those in the other two groups (Fig. 4). Additionally, positive cells were mostly located on the basal layer, as revealed by immunohistochemical staining. The MODs of the AOIs of four cell surface markers (CD71, CK14, integrin α6, and PCNA) in the DPSC group were significantly higher than those in the other two groups (Fig. 5).

### Improvements in the histological and inflammatory signs of radioactive esophageal injury following the infusion of DPSCs

**Fig. 6:**
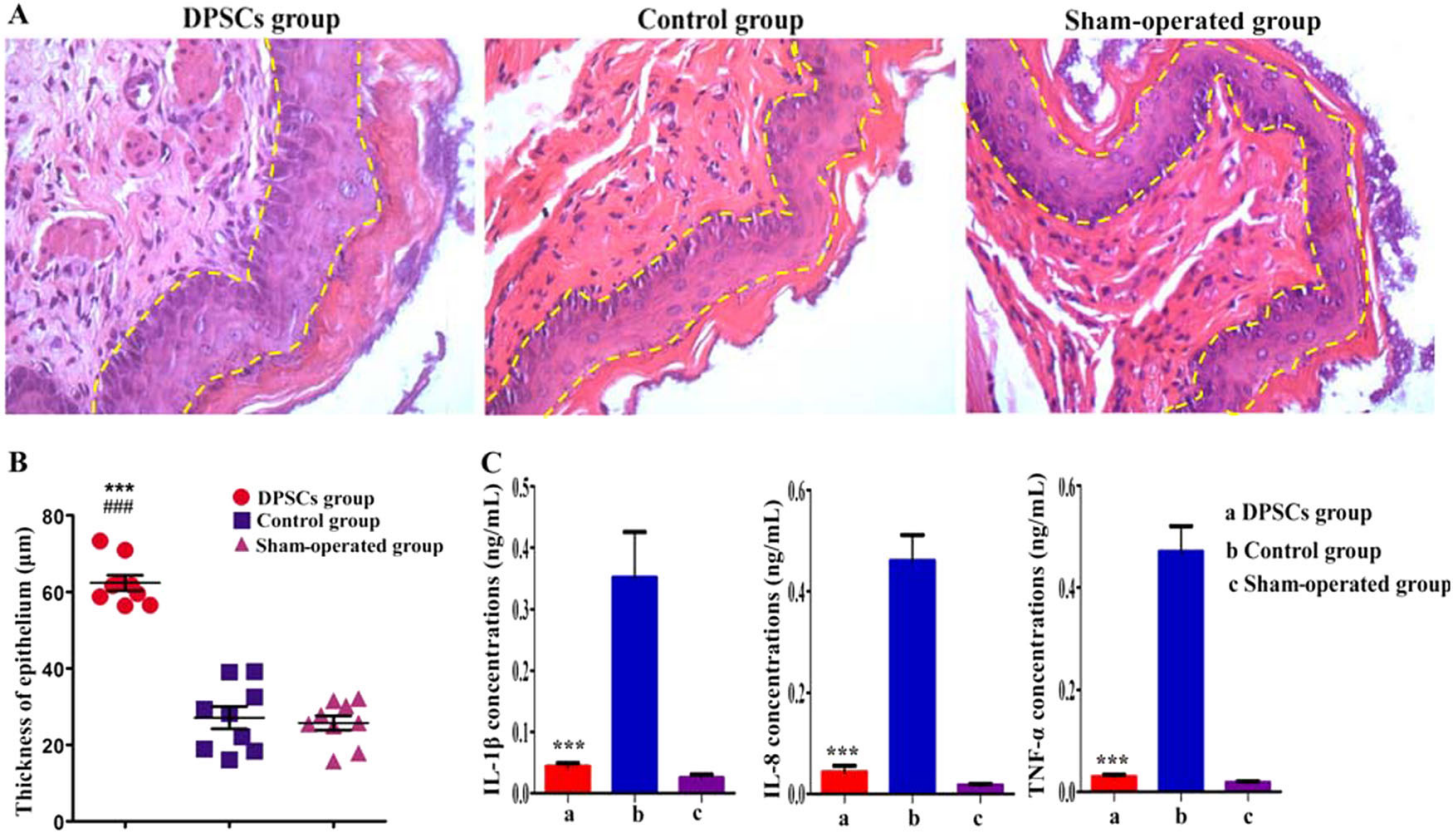
Histological and inflammatory improvements in radioactive esophageal injury with infused DPSCs. **a**, **b** The histological images of the esophagus revealed that the thickness of the epithelium in the DPSC group was significantly increased compared with those in the control and sham-operated groups (×400). **c** The TNF-α, IL-1β, and IL-8 concentrations in the DPSC group were not significantly different from those in the sham-operated group (*P* > 0.05). The TNF-α, IL-1β, and IL-8 concentrations in the control group were higher than those in the DPSC and sham-operated groups (*P* < 0.0001). *P < 0.05, **P < 0.001, and ***P < 0.0001, the DPSC group compared with the control group. ^#^*P* < 0.05, ^##^*P* < 0.001, and ^###^*P* < 0.0001, the DPSC group compared with the sham-operated group

The thickness of the esophageal epithelium from the same levels of the rat esophageal segments were measured and compared among the DPSC group, the control group, and the sham-operated group (Fig. 6a). The mean epithelial thickness was significantly increased in the DPSC group (62.35 ± 1.99 μm) relative to the control group (27.11 ± 2.92 μm) and the sham-operated group (25.71 ± 1.90 μm; Fig. 6b). The IL-1β, IL-8, and TNF-α levels in the DPSC group were not significantly different from those in the sham-operated group (*P* > 0.05). However, the TNF-α, IL-1β, and IL-8 levels in the control group were higher than those in the DPSC and sham-operated groups (*P* < 0.0001; Fig. 6c).

### Improvement in the esophageal function

**Fig. 7:**
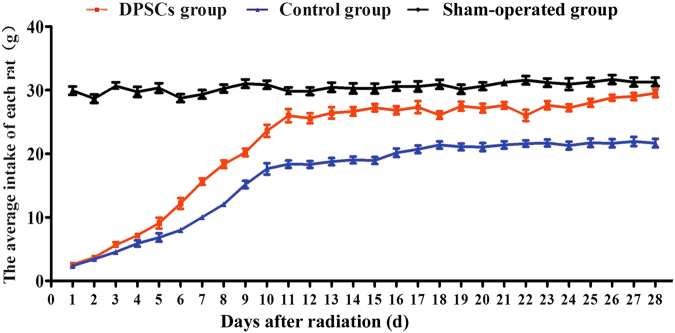
Average daily food intake levels of rats in all groups. The rats with DPSC treatment showed better food intake recovery; the food intake in the DPSC group was significantly higher than that in the control group from the sixth day forward, and the food intake in the DPSC group gradually approached the normal level

The DPSC and control groups basically underwent passive feeding and exhibited a worse condition and were less active than the sham-operated group after the ^125^I seeds were removed. The average intake of each rat was analyzed daily. Over time, the food intake levels of every rat in the DPSC and control groups gradually increased, and there was a significant difference between the DPSC and control groups beginning on the sixth day. The recuperative extrema of food intake in the DPSC group was significantly higher than that in the control group (*P* < 0.0001; Fig. 7).

## Discussion

After irradiation, the esophageal mucosa undergoes hyperemia and edema, leading to a series of complications, such as dysphagia, local pain, and retrosternal burning sensations^2^. These symptoms in turn can cause inadequate food intake, unbearable pain, malnutrition, and electrolyte disorders. Eventually, patients must stop radiotherapy before the duration recommended for optimal recovery is achieved^28^. Both supportive and symptomatic therapies are traditionally used to treat these complications. However, as SC research advances in the medical field, SC transplantation for treating radiation-induced esophageal damage has been explored as a new research direction. DPSCs are capable of homing, proliferation, and differentiation in injured tissue^23,29,30^.

In the present study, the SC source was DPSCs. Isolated DPSCs must be expanded before the SCs are transplanted. In the present study, most of the expanded DPSCs expressed CD90 and CD29, whereas few expressed CD45 and CD34. This result meets the defined standard of DPSCs established in previous studies^31^.

According to Epperly^13^, bone marrow cells also have the capacity to engraft on and differentiate into squamous cells in the irradiated esophagus. Although bone marrow cells and DPSCs display a similar capacity for differentiation, harvesting SCs from extracted teeth presents significant advantages compared with harvesting cells from bone marrow^32,33^ and the harvesting of other adult SCs that require more invasive procedures. DPSCs can be easily obtained from wisdom teeth, and there are minimal ethical issues involved in the use of DPSCs. Hence, tooth banking is an important step in future tissue engineering. Given these advantages, this study chose to investigate the use of DPSCs^18–20^. Meanwhile, SCs were transplanted via a vein in this study; this method has the advantage of being simple, widely applicable, safe, feasible, and highly effective. Directing the site-specific differentiation of DPSCs demands an accurate animal model, and the previous methods used to create animal models mainly relied on external exposure^34,35^ and the use of a higher radiation dose rate to administer a large amount of radiation to the radiation field within a short period. The irradiation was large for these rats, and the mortality was high. The exposed fields in the conventional methods were the neck and chest; thus, injury to the surrounding tissues was inevitable, so the in vitro model was not accurate. Therefore, we designed a new model in which ^125^I seeds placed in a disposable ureteral catheter were applied to irradiate the esophageal lumen in vivo. Since the disposable ureteral catheter could induce physical side injury to the esophageal cavity during the process, a sham-operated group was designed in which only the catheter lacking ^125^I seeds was inserted into the esophagus. Rats in the sham-operated group showed well-organized tissues and good food intake, similar to the normal group in the present study; thus, the possibility of catheter-induced damage was excluded. In the acute radiation esophagitis model (the model group) used in the present study, we observed radioactive damage similar to that described in previous studies^36^. In this model, which was induced by ^125^I seeds in vivo, the lumen of the esophagus was first irradiated, and the mucosa developed congestion, edema, inflammatory effusion, and exfoliation; meanwhile, inflammatory cytokines, including TNF-α, IL-1β, and IL-8, were released. The epithelium became thin, necrotic, and denuded. These changes induced dysphagia, odynophagia, and retrosternal pain. These symptoms and pathological changes are similar to those of acute clinical radioactive esophagitis^1,2,37^. ^125^I seeds provide low doses of radiation that quickly decrease with increasing distance^38^. The outer membrane displayed less damage in this model; hence, stricture and sclerosis were rarely observed. Moreover, the ^125^I seeds caused less damage to surrounding tissues over a short time. ^125^I seeds are widely used clinically and are easily obtained. ^125^I seeds as the low dose rate radioactive source are relatively safe for the rats with a low mortality rate^37,39,40^. These advantages allowed us to perform irradiation in vivo using this technique.

PKH26, a red fluorescent dye that labels the SC membrane, has a fast dyeing speed, stable properties, and no cytotoxicity^41^. PKH26 also glows red under a 551-nm excitation light, is observable using a fluorescence microscope, and is allocated to the daughter cells after DPSC differentiation. In the present study, DPSCs that were amplified in vitro were effectively labeled with PKH26 and were tracked in the rats’ bodies after transplantation.

The transplantation of DPSCs appeared to relieve the impairments in esophageal function in the rats with acute radioactive esophageal injury, as indicated by their increased food intake. The food intake of the DPSC group was significantly increased compared with the control group beginning on the sixth day. Similar to the functional improvement of acute radioactive esophageal injuries in rats treated with DPSCs, the same treatment significantly improved the radioactive damage to the esophageal epithelium. In addition, the degree of inflammation was significantly relieved in the DPSC group, as evidenced by the TNF-α, IL-1β, and IL-8 levels. After irradiation, the cells may release a malignant signal or chemical substance that stimulates DPSC homing and engraftment onto the injured esophageal tissue through the blood circulation. Then, the trans-differentiation and proliferation of the DPSCs increase the thickness of the esophageal epithelium^42,43^. These results provide new evidence of the therapeutic efficacy of DPSCs for treating acute radioactive esophageal injury. In the DPSC-treated rats, transplanted PKH26-labeled cells were found in the injured areas of the esophagus. These cells co-localized with PCNA, a nuclear antigen related to and synthesized during the cell cycle^44^, indicating that they proliferated in the esophagus. Furthermore, the transplanted DPSCs expressed CD71 and integrin α6, two cell surface markers used to identify putative SCs in the rat epidermis^45,46^. As shown in the study by Croagh et al.^45^, the α^bri^CD^bri^ cells represent a transit-amplifying population. These cells were the direct progeny of SCs, as they became a pool of dividing cells that increased to create differentiating suprabasal compartments. Moreover, the transplanted DPSCs co-localized with CK14, a squamous epithelial marker^47^. Based on the immunofluorescence (IF) staining, the transplanted DPSCs homed to the injured esophagus, where they may have proliferated and trans-differentiated into the esophageal SCs that repaired the injured mucosa of the DPSC-treated rats in vivo; the few PKH26-positive cells that were found in the spleen, lung, and liver indicated the slight injury in the tissue surrounding the esophagus. In the present study, the WB and immunohistochemical analyses revealed significantly higher expression levels of cell surface markers, including PCNA, CD71, integrin α6, and CK14, in the DPSC-treated group than those in the control and sham-operated groups. Thus, the transplanted DPSCs proliferated and trans-differentiated into mature esophageal epithelial cells in vivo and repaired the damaged esophageal tissue in the DPSC group. Some inflammatory factors were induced by and mediated primary esophageal epithelial proliferative responses to injury and regulated the healing of esophageal injuries in the control group; hence, the trans-differentiated cell makers were upregulated in the control group compared with those in the sham-operated group.

An obvious therapeutic effect of DPSC transplantation was observed on the 28th day in the present study. We observed the early behavior of DPSCs on the 7th, 14th, and 21st days; SC homing was found, but the trans-differentiation of DPSCs was not detected. The observations were extended to the 28th day, and trans-differentiation was widely detected. However, the experimental duration was too short to identify long-term complications of SC transplantation, including any carcinogenic changes. Further experiments are required to examine these complications in the future.

## Conclusions

After expansion in vitro, transplanted DPSCs recognize the radioactive injury and home to the injured esophagus, where the cells proliferate and trans-differentiate into esophageal SCs to repair the damaged esophageal tissue in vivo. The interaction between DPSCs and their niche, especially the involvement of signaling pathways, requires further research. Based on their regenerative performance, DPSCs heal tissue damage and improve the esophageal function following acute radioactive esophageal injury in rats. The findings of the present study imply that DPSC transplantation is an alternative approach for the treatment of acute radioactive esophageal injury. We encourage further preclinical studies and clinical trials of the potential use of DPSC transplantation in patients with acute radioactive esophageal injury. Although most patients with acute radioactive esophageal injury originally had a malignant tumor, researchers have not determined whether DPSC transplantation might increase the risk of recurrence and treatment difficulties. The best time to prevent or treat acute radioactive esophageal injury and the use of individualized therapy also requires further research and discussion.

## Materials and methods

### Experimental animals

Thirty-three adult male S–D rats weighing 280–300 g and two additional male rats weighing approximately 200 g were obtained from the Animal Facility of the Second Affiliated Hospital of Harbin Medical University, China. The animals were maintained in rooms at a constant temperature and humidity (23 ± 1 °C, 75%) with a 12/12-h light–dark cycle. The rats were then sterilized and given normal laboratory rat food ad libitum. All experimental animal protocols were approved by the Harbin Medical University Animal Care and Use Committee. All animals were treated under appropriate conditions according to the National Institute of Health Guidelines for the Care and Use of Experimental Animals.

### The isolation, culture, and differentiation of DPSCs

DPSCs were derived from both the upper and lower incisors of two adult male S–D rats weighing approximately 200 g after euthanasia. The dental pulp tissues were removed and suspended in phosphate-buffered saline (PBS) including 0.1% collagenase and 0.25% trypsin-ethylenediaminetetraacetic acid. DPSCs were cultured in alpha modified Eagle’s medium (GIBCO Laboratories, Inc., Grand Island, NY, USA) supplemented with 5.5 mmol/L glucose and 20% fetal bovine serum (FBS, Shanghai Solarbio, Shanghai, China) in an incubator at 37 °C with saturated humidity and 50 mL/L CO_2_. DPSCs of the first passage (P1), third passage (P3), and fourth passage (P4) in a good state were cultivated in 24-well culture plates at a density of 1 × 10^9^ cells/mL. The cell numbers were counted daily using a hemocytometer. Three wells were counted each day, the average numbers were calculated, and the values were used to plot a growth curve. The OriCell^TM^ osteogenesis differentiation kit (Cyagen, Guangzhou, China) was used to induce osteogenic differentiation. P3 SCs were cultured in osteogenic differentiation medium and fed every 3 days for 3 weeks. The alizarin red working solution was used to stain the cells for 3 to 5 min for alizarin red staining. Adipogenesis was induced by using the OriCell^TM^ adipogenic differentiation kit (Cyagen). The Oil Red O working solution (3:2 dilution with distilled water, filtered) was used to stain P3 SCs cultured in adipogenic differentiation medium for 30 min for Oil Red O staining. DPSCs (P3) were labeled with the fluorescent dye PKH26 (Sigma Aldrich, USA) following the manufacturer’s instructions^48^.

### The experimental esophageal model and study design

**Fig. 8:**
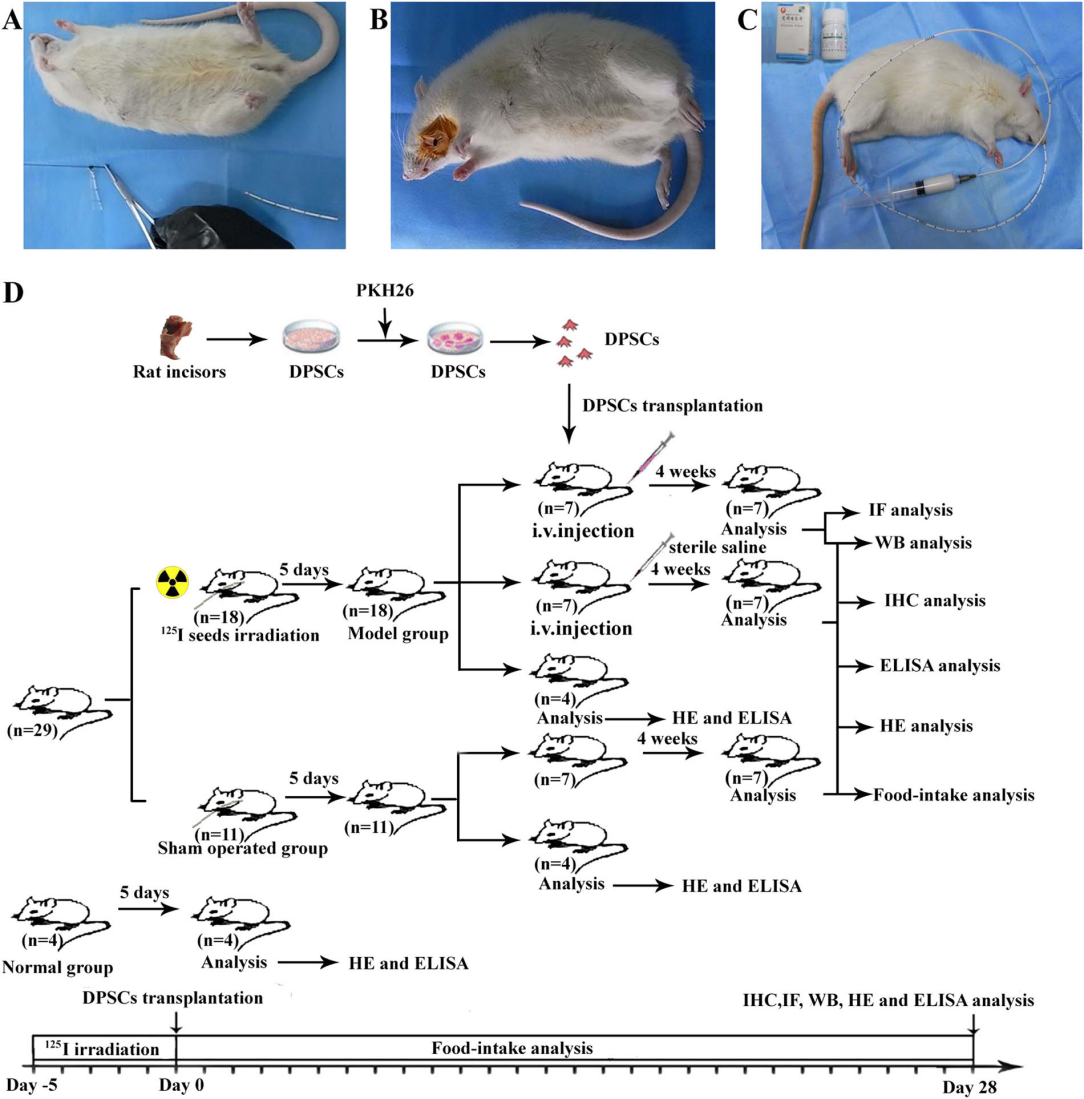
The steps to produce an in vivo model. **a** Five ^125^I seeds were placed close together in a disposable ureteral catheter, and the end of the catheter was stitched with a suture to secure the ^125^I. **b** The disposable ureteral catheter including the ^125^I seeds was fixed in the oral cavity. **c** The rats were administered food and estazolam through a disposable ureteral catheter when they were irradiated. **d** The flow chart of this study

Four of the 33 S–D rats from the normal group were randomly selected and omitted from any handling or procedures. Eighteen rats were then randomly selected from the remaining group and were irradiated using ^125^I seeds (Beijing Atom Technology Co., Ltd., 6711, China) with an initial activity of 0.8 mCi for 5 days. Next, the rats were anesthetized with 10% chloral hydrate (0.3 mL/100 g, Gongsi) via intraperitoneal injection and were fixed on the board in the supine position. Five ^125^I seeds were placed close together at a depth of 8 cm in a disposable ureteral catheter (Shanghai Kangge Medical Equipment Co., Ltd., production batch number: 101103, Specification: F4, China) using a guidewire passed through the oral cavity into the esophagus (Fig. 8a). The catheter was fixed in the oral cavity with a suture after the guidewire was removed (Fig. 8b). X-rays were obtained after the operation to confirm whether the ^125^I seeds were located in the rat’s esophagus. The remaining rats were assigned to the sham-operated group, in which only a disposable ureteral catheter was placed into the esophagus. The rats (*n* = 29) were fed liquid diets through the catheter when they were awake. During feeding, the rats were also administered estazolam via the catheter to calm them (Fig. 8c). Four rats were randomly chosen from every group, euthanized on the fifth day, and the radiation injury was evaluated. The rats in the radioactive esophageal injury model group were randomly divided into two groups of seven rats each. The animals in the experimental group were injected with DPSCs (1 × 10^7^ cells) via the tail vein, whereas the control group received sterile saline. The rats were regularly provided with food and water daily, and the food intake was assessed daily. Three groups of rats were euthanized on the fourth week after transplantation, and their esophageal tissues were removed to evaluate their recovery (Fig. 8d).

### Flow cytometry

FACS was used to analyze the expression levels of the various antigen markers of cultured DPSCs^49^. SCs (1 × 10^6^) were incubated with 1 μL of rat monoclonal antibodies against CD90, CD29, CD45, and CD34 (BD Bioscience, San Jose, CA; 1 μL) in 2% FBS at 4 °C for 30 min. Cells from the negative control group were incubated in the buffer without any major antibodies. The fluorescence signals were analyzed using FACSCalibur Cell Quest software (Becton Dickinson, USA).

### Tissue preparation

On the estimated date after irradiation and transplantation, the rats were sacrificed by myocardial perfusion with 4% paraformaldehyde dissolved in PBS after anesthesia with 10% chloral hydrate (intraperitoneally). The upper two-thirds of each esophagus were removed from the thorax and abdomen, rinsed with PBS, and immersed in 30% sucrose overnight at 4 °C. The esophageal tissue was embedded in optimum cutting temperature compound (OCT) after freezing, prepared into frozen sections with a thickness of 6 μm, and mounted onto glass slides. The histological evaluation included hematoxylin and eosin (HE) staining of the first and last cuts of the serial sections, and sections selected at intervals of 20 were used for IF and immunohistochemical (IHC) staining.

### Immunohistochemistry

IHC staining was performed as described previously^50^. Sections were submerged in 0.3% hydrogen peroxide methanol solution for 30 min, incubated in PBS containing 5% normal horse serum and 0.3% Triton X-100 for 2 h, and then incubated with PCNA, CK14, integrin α6, and CD71 primary antibodies overnight at 4 °C. Sections were then kept at room temperature (RT) for 40 min and treated with pre-diluted biotinylated anti-mouse immunoglobulin secondary antibody for 2 h and avidin biotin complex reagent (1:400) for 1 h. Immunoreaction was visible under 0.05% diaminobenzidine and 0.003% H_2_O_2_. The sections were washed three times after incubation (10 min per wash), dried, dehydrated with ethanol gradient, washed with dimethylbenzene, mounted, and baked.

### Histological and IF staining

The esophageal histological analysis using HE staining followed the established standard procedures^51^. IF staining was performed to examine the homing and differentiation of infused DPSCs in the esophageal tissue^52^. Sections of the esophagus were incubated in PBS containing 5% normal donkey serum and mouse anti-PCNA antibody (1:100, Santa Cruz Biotechnology, BM0104), mouse anti-CK14 antibody (1:100, Abcam, ab49747), mouse anti-integrin α6 antibody (1:100, Santa Cruz Biotechnology, sc-13542) and mouse anti-CD71 antibody (1:200, AbD, MCA155R) overnight at 4 °C. Sections were then washed with PBS and subsequently reacted for 1 h with goat anti-mouse IgG (H + L; 1:200, Earthox, E032220) in a box with a constant temperature of 37 °C. Sections were re-dyed with DAPI (Vector Laboratories, California, USA), washed, and mounted with an anti-fading medium before undergoing microscopic observations with a laser confocal microscope.

### Western blotting

The esophageal tissues were homogenized in lysis buffer containing proteinase inhibitors (Jianglai Biotech, Shanghai, China)^53^. The lysates were centrifuged at 12,000 rpm for 10 min at 4 °C, and the concentrations of the proteins were determined with a protein kit (Bio-Rad Laboratories Inc., Hercules, CA, USA). Twenty micrograms of each esophageal protein was electrophoresed on a 5% sodium dodecyl sulfate-polyacrylamide gel and then transferred to polyvinylidene difluoride membranes (Millipore, Bedford, MA, USA). The membranes were blocked with 5% skim milk in Tris-buffered saline including 0.1% Tween-20 for 1 h at room temperature. The membranes were then incubated with primary anti-CD71 (Santa Cruz Biotechnology; 1:200), anti-CK14 (Abcam; 1:100), anti-PCNA (Santa Cruz Biotechnology, 1:100), or anti-integrin α6 (Santa Cruz Biotechnology, 1:100) antibodies overnight at 4 °C and subsequently incubated with horseradish peroxidase-conjugated secondary antibodies for 1 h at room temperature. Specific protein bands were developed using an enhanced chemiluminescence detection kit (Amersham, Piscataway, NJ, USA). The membranes were probed with β-actin antibody (Abcam), and the intensity of each protein band was normalized to β-actin.

### Enzyme-linked immunosorbent assay

Two milliliters of blood drawn from the rat’s tail vein was centrifuged at a rate 3000 r/min, and the serum was separated. The concentrations of IL-1β, IL-8, and TNF-α were tested using enzyme-linked immunosorbent assays (eBioscience)^54^.

### Statistical analysis

The data are expressed as the mean ± SEM. Student’s *t* test and the nonparametric Mann–Whitney *U* test were used to assess the differences, and *P* < 0.05 was defined as a significant difference.
